# Physical fitness and psychosocial health in a sample of Dutch adolescents

**DOI:** 10.1016/j.pmedr.2021.101689

**Published:** 2021-12-27

**Authors:** Barbara Franca Haverkamp, Esther Hartman, Jaap Oosterlaan

**Affiliations:** a University of Groningen, University Medical Center Groningen, Center for Human Movement Sciences, Groningen, the Netherlands; b Vrije Universiteit Amsterdam, Faculty of Behavioural and Movement Sciences, Clinical Neuropsychology Section, Amsterdam, the Netherlands; c Emma Children’s Hospital, Amsterdam UMC, University of Amsterdam, Departmct of Pediatrics, Emma Children’s Hospital Amsterdam UMC Follow-Me Program & Emma Neuroscience Group, Amsterdam Reproduction & Development, Amsterdam, the Netherlands

## Abstract

Adolescence is characterized by profound changes in body and behavior, and not surprisingly during this developmental phase the risk of developing psychosocial problems increases dramatically. The purpose of the current study was to examine the relationship of both physical fitness and body composition with psychosocial health in adolescents (12–15 years). Data were collected in 2019–2020 in a representative sample of 361 Dutch adolescents (46.3% boys, age = 13.44 ± 0.43 years). Physical fitness and body composition were assessed by subtests of the Eurofit test battery assessing cardiorespiratory fitness (20 m Shuttle Run Test), muscular fitness (Broad Jump and Sit-Ups), speed-agility (10x5-m Shuttle Run Test and Fast Tapping Test), and body composition (Body Mass Index). Psychosocial health was assessed in four domains: self-concept (Competence Experience Scale for Adolescents), symptoms of depression (Child Depression Inventory), anxiety (State and Trait Anxiety Inventory) and ADHD (Strengths and Weaknesses of ADHD Symptoms). Multilevel regression analyses were performed in MLwin. Results showed that better cardiorespiratory fitness was related to better self-concept (β = 0.225; p < 0.001), less symptoms of depression (β = −0.263; p = 0.003), and lower levels of state (β = −0.239; p = 0.008) and trait anxiety (β = –232; p = 0.008). Furthermore, higher BMI was related to lower self-concept (β = −0.075; p = 0.019). Taken together, the results suggest that better cardiorespiratory fitness and lean body composition have a positive relationship with self-concept and that better cardiorespiratory fitness is related to less symptoms of depression and anxiety.

## 1 Introduction

Adolescence has been noted as a period of dramatic changes in body and behavior (Paus et al., 2008). Changes in the neural system subserve higher cognitive functions, reasoning and interpersonal interactions, cognitive control of emotions, risk-versus-reward appraisal and motivation. These changes are usually beneficial and optimize the brain for the challenges ahead, but they can also confer a vulnerability to certain types of psychopathology. The emergence of certain psychopathologies is probably related to anomalies or exaggerations of typical adolescent maturation processes acting in concert with psychosocial factors (for example, school and relationships) and/or biological environmental factors (for example, drugs or alcohol abuse) (Paus et al., 2008). Psychosocial health (i.e. mental health) is a multidimensional state of well-being, with both negative indicators, such as depression, anxiety or behavioral problems, and positive indicators, such as self-concept (Dale et al., 2019). Around 10–20% of the adolescents suffer from psychosocial health problems, with subclinical psychosocial health problems being more prevalent (Viner and Booy, 2005). Depression, suicidal thoughts and actions, eating disorders, anxiety as well as continuing childhood problems, such as attention-deficit hyperactivity disorder (ADHD), are some of the conditions that affect adolescents at disproportionate rates compared to other age groups (Viner and Booy, 2005). Furthermore, psychosocial health problems during adolescence are a strong risk factor for persisting psychopathologies later in life (Paus et al., 2008). These findings emphasize the importance of finding effective methods to cope with, prevent or improve adolescents’ psychosocial health.

Physical activity has been related to better self-concept, and has also been suggested to be a protective factor against psychosocial health problems such as depression, anxiety, and ADHD (Dale et al., 2019, Biddle et al., 2019, Vysniauske et al., 2020). While physical activity is moderately related to physical fitness, there are also other factors that influence physical fitness such as genetics, biological maturation, nutrition, as well as the physical and social environment (Raghuveer et al., 2020, Bacil et al., 2015). In this study we investigate the relationship between physical fitness and psychosocial health. Physical fitness refers to the ability to engage in physical activity for a protracted period of time (Martínez-Vizcaíno and Sánchez-López, 2008) and reflects a proxy of the past type, intensity, frequency and duration of physical activity. Physical fitness may be defined in terms of body composition, cardiorespiratory fitness, muscular fitness, flexibility, balance, coordination, and speed-agility (Ortega et al., 2008, Corbin et al., 2000). The main components cardiorespiratory fitness, muscular fitness, speed-agility and body composition will be analyzed in this study (Ortega et al., 2008).

Lubans has proposed three mechanisms explaining the effects of physical activity on psychosocial health (Lubans et al., 2016). Although these mechanisms pertain to the effects of physical activity, these mechanisms likely also apply to the effects of physical fitness on psychosocial health as physical activity is an important determinant of physical fitness. First, at the neurobiological level, physical activity promotes an increase of cerebral blood flow, synaptic plasticity and brain-derived neurotrophic factor levels that support structural and functional changes in the brain (Dishman and O'Connor, 2009). Furthermore, participation in physical activity has been found to lead to higher concentrations of endorphins in the brain and to reduce activity of the sympathetic nervous system, resulting in less cortisol and in turn resulting in less internalizing problems in adults and adolescents (Anderson and Shivakumar, 2013, Dinas et al., 2011, Mücke et al., 2018). Second, at the psychosocial level, physical activity has the potential to improve well-being via a range of mechanisms: It provides an opportunity for social interaction, as well as improvements in appearance, physical self-perception and independence (autonomy), all leading to improved psychosocial health (Lubans et al., 2016, Lubans et al., 2016). Third, at the behavioral level, physical activity might influence self-regulation and coping skills that have subsequent implications for psychosocial health outcomes (Lubans et al., 2012). For example during physical education lessons or sports participation adolescents have the possibility to practice self-control skills, conflict-resolution and to learn cooperating with others, and to develop honesty, fairness, and moral judgement that might foster psychosocial health outcomes (Lubans et al., 2016, Eime et al., 2013). Finally, physical activity provokes changes in body composition which in turn may have beneficial effects on psychosocial health via changes in others’ attitudes towards the person in question (Russell-Mayhew et al., 2012). It is suggested that others’ attitudes and weight based stigmatization and teasing, may cause weight and shape concerns in adolescents that in turn negatively impact on psychosocial health (Russell-Mayhew et al., 2012).

Some previous studies investigated the relationship between physical fitness and the broad concept of psychosocial health, whereas others studied specific aspects of psychosocial health, such as anxiety and depression, or self-concept. For example, in a study of Janssen et al., (Janssen et al., 2020) cardiorespiratory fitness was positively related to mental health, and muscular fitness was not. Furthermore, Åvitsland et al. found a positive relationship of cardiorespiratory fitness and muscular fitness with overall psychosocial health, but the relationship between body composition and psychosocial health failed to reach significance (Åvitsland et al., 2020). However, when all aspects of physical fitness were entered together in one model, only cardiorespiratory fitness was found to be significantly related to psychosocial health. Furthermore, healthier body composition and higher levels of cardiorespiratory fitness have been related to lower levels of depression in adolescents (Bou-Sospedra et al., 2020, Rieck et al., 2013, Mannan et al., 2016). However, no significant relationship was found between muscular fitness or speed-agility and depression (Bou-Sospedra et al., 2020). Furthermore, Cadenas-Sanchez et al. (Cadenas-Sanchez et al., 2021) showed in their review that cardiorespiratory fitness was related to better self-concept and less symptoms of depression. In addition, they showed that muscular fitness was related to better self-concept and less symptoms of anxiety. Taken together, the previous research that has been conducted in adolescents has suggested that there is a positive relationship between physical fitness and psychosocial health, however, it also revealed inconsistent evidence and more research is clearly needed to clarify the relationship between aspects of physical fitness and psychosocial health.

The aim of the current study was to examine the relationship of aspects of physical fitness (cardiorespiratory fitness, muscular fitness, speed-agility and body composition) with aspects of psychosocial health (self-concept, and symptoms of depression, anxiety and ADHD) in adolescents. Based on previous literature in adolescents and other populations, we hypothesized that better cardiorespiratory fitness, muscular fitness, and speed-agility, and lower body composition would be associated with better psychosocial health.

## 2 Methods

### 2.1 Participants

A total of 361 adolescents (194 girls, 167 boys, ages 12–16 years) were recruited from seven secondary schools in the northern part of the Netherlands. To obtain a representative sample, we included adolescents from both non-urban and urban schools, different educational levels. Adolescents and their parents/caregivers were informed about the study by an information letter and written informed consent was obtained prior to participation. Demographics of the participating adolescents are shown in Table 1.

**Table 1:** Participant characteristics (N = 361).

Age, M (SD), years		13.44 (0.430)
Sex, n girls (%)		194 (53.7%)
BMI		
	Healthy weight, n (%)^a^	316 (87.5%)
	Overweight, n (%)^a^	34 (9.4%)
	Obese, n (%)^a^	11 (3.0%)
Educational level		
	Prevocational secondary education, n (%)	72 (19.9%)
	Higher general secondary education, n (%)	162 (44.9%)
	Pre-university education, n (%)	127 (35.2%)

*Note.* BMI, body mass index; ^a^According to the reference values by Cole et al. (Cole et al., 2000).

### 2.2 Measurement instruments

#### 2.2.1 Physical fitness

Physical fitness was assessed using the Eurofit test battery (Adam et al., 1993). From this test battery, six subtests were assessed by well-trained examiners using standardized instructions. Vrijkotte et al. (Vrijkotte et al., 2007) reported good reliability (r > 0.75) and satisfactory validity for the Eurofit test battery in adolescents aged 12–16 years. Cardiorespiratory fitness was measured with the 20-m Shuttle Run Test. Running speed started at 8 km.h^−1^ and increased every minute with 0.5 km.h^−1^. The test ended when an adolescent failed to reach a line prior to the audio signal on two successive trials. The peak VO_2_ was computed with the equation proposed by Barnett et al. (Barnett et al., 1993). Muscular fitness was measured by Sit-Ups and Broad Jump. The amount of Sit-Ups performed within 30 s was recorded as a test result. For Broad Jump the longest distance jumped (in cm) was recorded as the test result. Speed-agility was measured by the 10x5-m Shuttle Run Test and the Plate Tapping Test. Time needed to run the 10x5-m Shuttle Run Test was recorded. During the Plate Tapping Test adolescents needed to consecutively touch two discs, that were 80 cm apart, as fast as possible. The time needed to complete 25 full cycles was recorded. The best of two attempts was used as a test result in both tests. Body composition was assessed by the Body Mass Index (BMI). Participants’ weight, without shoes, was measured by digital scale, that was calibrated after each measurement week and all measurements were recorded to the closest 0.1 kg. Height was measured by tape-measure, without shoes, and all measurements were recorded to the closest cm. BMI scores (kg/m^2^) were calculated by dividing weight by height squared.

#### 2.2.2 Psychosocial health

Psychosocial health was assessed using self-reported questionnaires of self-concept, and symptoms of depression, anxiety and ADHD. Self-concept was assessed with the Competence Experience Scale for Adolescents (CBSA) (Treffers et al., 2002), which is based on Self Perception Profile for Adolescents (Harter, 1978). The CBSA consists of seven subscales: School Skills, Social Acceptance, Athletic Competence, Physical Appearance, Behavioral Attitude, Close Friendships, and Self-Esteem. Each subscale consists of five items that are rated in a four-point scale, with scores ranging from 1 to 4 points. Raw scores were calculated by summing scores on all items of each subscale, resulting in a raw score between 5 and 20 points per subscale, with higher scores indicating better self-concept. Adequate reliability and validity have been reported for the questionnaire (Treffers et al., 2002).

The Children’s Depression Inventory (CDI; (Kovacs and Beck, 1977) was used to assess symptoms of depression. The CDI consisted of 27 items that are scored on a three-point Likert scale (scores 0–2 points). A raw score was calculated by summing scores on all items, resulting in a score between 0 and 54 points, with higher scores indicating more severe symptoms of depression. Adequate reliability and validity have been reported for the CDI (Kovacs and Beck, 1977, Smucker et al., 1986).

The State- Trait Anxiety Inventory (STAI; (Spielberger, 1983) was used to assess state and trait anxiety symptoms and contains two scales each comprising 20 items scored on a three-point Likert scale (scores 0–2 points). Raw scores on the two scales were calculated by summing scores on all items of each scale, resulting in a raw score between 0 and 40 points, with higher scores indicating higher levels of anxiety. The STAI has shown adequate reliability (Spielberger, 1983).

Symptoms of ADHD were measured using the Strengths and Weaknesses of ADHD symptoms and Normal behavior rating scale (SWAN). The SWAN contains two scales of nine items, one measuring Inattentive Behavior and the other measuring Hyperactive-Impulsive Behavior. Items are scored on a seven-point Likert scale (scores 1–7 points), with scores < 4 indicating higher levels of Inattentive, and Hyperactive-Impulsive Behaviors as compared to same aged peers (Brites et al., 2015). The average score of the nine items was used as raw test score.

### 2.3 Procedure

Adolescents were tested during two physical education lessons within the same week. In the first lesson, the 20-m Shuttle Run Test was administered and height and weight were measured. In the second lesson, the remaining physical fitness tests were administered. In the same week, mental health questionnaires were completed in the classroom under supervision of a teacher and research assistant. The study procedures were in accordance with and approved by the medical ethical committee of the University Medical Center Groningen, the Netherlands. The current data were obtained as part of a larger study registered in the Dutch Trial Register (# NTR7098).

### 2.4 Data analysis

Initial analyses were performed in IBM SPSS Statistics version 26.0. Outliers (z ≤ −3.29 or z ≥ 3.29) were winsorized (Tabachnick et al., 2007). Values missing at random were imputed for a particular variable only if the following conditions were met: (1) < 20% of the data for that variable were missing, (2) there were sufficient data available (<40% data points missing) on other measures of physical fitness or psychosocial health to impute the missing data. Missing values were replaced by multiple imputation using the average of five imputed variables (Sterne et al., 2009). Non-normally distributed variables were converted using van der Waerden transformations. Measures of physical fitness and psychosocial health were transformed into z-scores that were used in subsequent all analyses. An average z-score was calculated for Sit-Ups and Broad Jump, representing muscular fitness. Likewise, the inverse average z-score was calculated for 10x5-m Shuttle Run Rest and the Fast Tapping Test, representing overall speed-agility. Finally, one average z-score was calculated for all domains of self-concept, representing overall self-concept. Higher scores reflected (1) better cardiorespiratory fitness, muscular fitness, speed-agility, and higher BMI, and (2) better self-concept, lower levels of symptoms of depression and anxiety, and lower levels of inattentive, and hyperactive-impulsive behaviors.

The main analysis comprised multivariate multilevel regression analysis (MLwiN, version 3) accounting for the variability between classes (e.g. educational level) and nesting of adolescents within classes. Psychosocial health outcomes were used as dependent variables in the models. A random intercept was used for each class (level 3). Two models were build. Model 1 contained only the covariate sex as predictor of the dependent variables. Model 2 extended model 1 by adding all aspects of physical fitness. The model fit was evaluated by comparing the deviance (−2*log-likelihood) of the first model and the second model using a χ^2^ difference test. If model 2 showed improved fit compared to model 1, the unique contribution of each of the entered predictors was determined. Finally, if a significant relationship between aspects of physical fitness and the overall measure of self-concept was obtained, follow-up analyses were conducted on each of the seven subscales of the CBSA. Level of significance was set at p < 0.05 (two sided). Effect sizes (ESs) were calculated as [(estimated effect of predictor B *2)/(√(1-B2)] (Tymms, 2004). An effect size below 0.20 is considered negligible, 0.20–0.49 small, 0.50–0.79 medium and above 0.80 strong (Cohen, 1988).

## 3 Results

The raw scores for physical fitness and psychosocial health are presented in Table 2.

**Table 2:** Raw test scores on measures of physical fitness and psychosocial functioning (N = 361).

Concept measured	Measure	Mean (SD)	Range
Cardiorespiratory fitness	VO_2_max (ml.kg^−1^.min^−1^)	47.53 (5.015)	35.36–59.37
Muscular fitness	Sit-Ups (number in 30 s)	19.66 (3.633)	10–31
	Broad Jump (cm)	158.46 (25.544)	93.0–251.0
Speed-agility	10×5-m Shuttle Run Test (s)	20.68 (1.865)	16.5–26.5
	Plate Tapping Test (s)	12.08 (1.268)	9.1–17.2
Body composition	Body Mass Index (kg/m^2^)	19.18 (3.349)	13.42–40.12
Self-concept	School Skills	13.44 (2.794)	5–20
	Social Acceptance	15.11 (2.773)	6–20
	Athletic Competence	13.75 (3.531)	5–20
	Physical Appearance	13.95 (3.679)	5–20
	Behavioral Attitude	15.52 (3.043)	5–20
	Close Friendships	17.16 (2.752)	6–20
	Self-Esteem	15.47 (3.226)	5–20
Depression symptoms	CDI	8.72 (6.133)	0–34
Anxiety symptoms	State Anxiety (STAI)	10.74 (5.037)	0–35
	Trait Anxiety (STAI)	10.42 (7.457)	0–40
ADHD symptoms	Inattentive Behavior (SWAN)	4.44 (0.781)	1.88–6.67
	Hyperactive-Impulsive Behavior (SWAN)	4.42 (0.966)	2.22–6.89

*Note.* CDI: Child Depression Index; STAI: State- Trait Anxiety Inventory; ADHD: Attention-Deficit Hyperactivity Disorder, SWAN: Strengths and Weaknesses of ADHD symptoms and Normal behavior rating scale.

Results revealed that model 2 had significantly better model fit than model 1, Δχ^2^ (Biddle et al., 2019) = 72.38, p < 0.001, indicating that adding aspects of physical fitness to model 1 (including random intercept and sex), increased the percentage of variance explained in the psychosocial health measures. Table 3 shows the results of the multivariate multilevel regression models testing the associations of the three aspects of physical fitness and body composition with the psychosocial health measures.

**Table 3:** Results of multivariate multilevel regression analysis for physical fitness and psychosocial health (n = 361).

	Model 1			Model 2		
	B	SE	p	B	SE	p
*Self-concept*						
Random intercept	0.054	0.051	0.289	−0.152	0.063	0.016
Sex^a^	−0.107	0.064	0.094	0.270	0.093	0.004
VO2max				**0.225**	**0.053**	**0.000**
Muscular fitness				0.038	0.045	0.399
Speed-agility				0.032	0.044	0.461
Body composition				**−0.075**	**0.032**	**0.019**
Variance classes^b^	0.008	0.007	0.253	0.012	0.008	0.133
Variance adolescents^b^	0.369	0.028	<0.001	0.319	0.024	<0.001
*Depression*						
Random intercept	−0.036	0.085	0.669	0.194	0.104	0.062
Sex^a^	0.083	0.104	0.423	−0.343	0.156	0.027
VO2max				**−0.263**	**0.088**	**0.003**
Muscular fitness				0.020	0.075	0.794
Speed-agility				−0.078	0.073	0.286
Body composition				0.104	0.054	0.052
Variance classes^b^	0.030	0.018	0.096	0.028	0.017	0.100
Variance adolescents^b^	0.963	0.073	<0.001	0.908	0.068	<0.001
*State Anxiety*						
Random intercept	−0.098	0.077	0.203	0.083	0.100	0.407
Sex^a^	0.182	0.105	0.083	−0.153	0.159	0.336
VO2max				**−0.239**	**0.090**	**0.008**
Muscular fitness				0.067	0.076	0.380
Speed-agility				0.022	0.074	0.771
Body composition				0.076	0.055	0.165
Variance classes^b^	0.000	0.000	1.000	0.000	0.000	1.000
Variance adolescents^b^	0.988	0.074	<0.001	0.958	0.072	<0.001
*Trait Anxiety*						
Random intercept	−0.257	0.075	0.001	−0.056	0.097	0.563
Sex^a^	0.479	0.102	0.000	0.104	0.154	0.499
VO2max				**−0.232**	**0.087**	**0.008**
Muscular fitness				−0.052	0.074	0.479
Speed-agility				0.053	0.072	0.464
Body composition				0.073	0.053	0.172
Variance classes^b^	0.000	0.000	1.000	0.000	0.000	1.000
Variance adolescents^b^	0.938	0.070	<0.001	0.899	0.067	<0.001
*Inattentive Behavior*						
Random intercept	−0.042	0.084	0.612	−0.091	0.105	0.389
Sex^a^	0.075	0.104	0.474	0.163	0.159	0.306
VO2max				0.073	0.090	0.415
Muscular fitness				−0.024	0.077	0.751
Speed-agility				−0.035	0.075	0.637
Body composition				−0.021	0.055	0.704
Variance classes^b^	0.026	0.024	0.279	0.025	0.024	0.298
Variance adolescents^b^	0.976	0.074	<0.001	0.972	0.074	<0.001
*Hyperactive and Impulsive Behavior*						
Random intercept	−0.069	0.093	0.458	−0.042	0.112	0.707
Sex^a^	0.147	0.104	0.156	0.093	0.159	0.558
VO2max				0.025	0.090	0.782
Muscular fitness				−0.131	0.076	0.086
Speed-agility				−0.089	0.075	0.236
Body composition				0.031	0.054	0.570
Variance classes^b^	0.065	0.037	0.079	0.058	0.034	0.088
Variance adolescents^b^	0.939	0.072	<0.001	0.922	0.071	<0.001
Deviance	4793.79			4721.41		

*Note*. ^a^Boys were used as the reference category; ^b^Values representing between and within class variance, respectively.

Next, we tested the univariate contribution of cardiorespiratory fitness, muscular fitness, speed-agility and body composition to the six psychosocial health measures in model 2 (see Appendix for full results). These results showed that better cardiorespiratory fitness was significantly related to better self-concept, t = 4.245, p < 0.001, ES = 0.461 as well as less symptoms of depression, t = 2.988, p = 0.003, ES = 0.263, state anxiety, t = 2.656, p = 0.008, ES = 0.492 and trait anxiety, t = 2.667, p = 0.008, ES = 0.477. Cardiorespiratory fitness was not significantly related to either inattentive or hyperactive-impulsive behavior. Furthermore, it was shown that body composition was significantly related to self-concept, t = 2.344, p = 0.019, ES = 0.150, with higher body composition being related to lower self-concept. Body composition was not significantly related to symptoms of depression, anxiety and ADHD. Furthermore, neither muscular fitness, nor speed-agility were significantly related to any of the psychosocial health outcomes.

Next, we tested the univariate contribution of cardiorespiratory fitness, muscular fitness, speed-agility and body composition to the seven aspects of self-concept using multivariate multilevel regression models (see Table 4 for full results). The results showed that better cardiorespiratory fitness was significantly related to better scores on the scales Social Acceptance, t = 4.128, p < 0.001, ES = 0.759, Athletic Competence, t = 6.013, p < 0.001, ES = 1.028, Close Friendships, t = 2.978, p = 0.003, ES = 0.549 and Self-Esteem, t = 2.690, p = 0.007, ES = 0.481. Cardiorespiratory fitness was not significantly related to school skills, physical appearance and behavioral attitude. Furthermore, it was shown that muscular fitness and speed-agility were significantly related better to Athletic Competence, t = 4.172, p < 0.001, ES = 0.554 and t = 2.429, p = 0.015, ES = 0.310, respectively. Finally, higher body composition (BMI) was significantly related to lower scores on Physical Appearance, t = 4.692, p < 0.001, ES = 0.503, and Self-Esteem, t = 3.321, p = 0.001, ES = 0.481. All other relationships were not significant.

**Table 4:** Results of multivariate multilevel regression analysis for physical fitness and self-concept (n = 361).

	Model 2		
	B	SE	p
*School Skills*			
Random intercept	−0.024	0.101	0.809
Sex^a^	0.046	0.161	0.778
VO2max	0.067	0.091	0.461
Muscular fitness	−0.065	0.077	0.400
Speed-agility	0.135	0.075	0.072
Body composition	0.012	0.056	0.834
Variance classes^b^	0.000	0.000	1.000
Variance adolescents^b^	0.984	0.073	0.000
*Social Acceptance*			
Random intercept	−0.137	0.095	0.150
Sex^a^	0.255	0.152	0.093
VO2max	**0.355**	**0.086**	**0.000**
Muscular fitness	0.115	0.073	0.114
Speed-agility	0.062	0.071	0.383
Body composition	0.030	0.052	0.565
Variance classes^b^	0.000	0.000	1.000
Variance adolescents^b^	0.875	0.065	0.000
*Athletic Competence*			
Random intercept	−0.240	0.085	0.004
Sex^a^	0.447	0.135	0.001
VO2max	**0.457**	**0.076**	**0.000**
Muscular fitness	**0.267**	**0.064**	**0.000**
Speed-agility	**0.153**	**0.063**	**0.015**
Body composition	−0.015	0.047	0.753
Variance classes^b^	0.000	0.000	1.000
Variance adolescents^b^	0.688	0.051	0.000
*Physical Appearance*			
Random intercept	0.063	0.095	0.505
Sex^a^	−0.118	0.152	0.437
VO2max	0.156	0.086	0.070
Muscular fitness	0.006	0.073	0.936
Speed-agility	−0.017	0.071	0.807
Body composition	**−0.244**	**0.052**	**0.000**
Variance classes^b^	0.000	0.000	1.000
Variance adolescents^b^	0.873	0.065	0.000
*Behavioral Attitude*			
Random intercept	−0.261	0.097	0.007
Sex^a^	0.486	0.154	0.002
VO2max	−0.029	0.087	0.735
Muscular fitness	−0.069	0.074	0.352
Speed-agility	−0.094	0.072	0.192
Body composition	−0.065	0.053	0.221
Variance classes^b^	0.000	0.000	1.000
Variance adolescents^b^	0.901	0.067	0.000
*Close Friendships*			
Random intercept	−0.294	0.099	0.003
Sex^a^	0.548	0.158	0.001
VO2max	**0.265**	**0.089**	**0.003**
Muscular fitness	−0.028	0.075	0.713
Speed-agility	0.034	0.073	0.642
Body composition	−0.096	0.054	0.076
Variance classes^b^	0.000	0.000	1.000
Variance adolescents^b^	0.942	0.070	0.000
*Self-Esteem*			
Random intercept	−0.022	0.097	0.820
Sex^a^	0.041	0.154	0.790
VO2max	**0.234**	**0.087**	**0.007**
Muscular fitness	−0.007	0.074	0.923
Speed-agility	−0.007	0.072	0.920
Body composition	**−0.176**	**0.053**	**0.001**
Variance classes^b^	0.000	0.000	1.000
Variance adolescents^b^	0.899	0.067	0.000
Deviance			

*Note*. ^a^Boys were used as the reference category; ^b^Values representing between and within class variance, respectively.

## 4 Discussion

The aim of the current study was to examine the relationship of aspects of physical fitness with psychosocial health in adolescents. The main findings of the present study showed that better cardiorespiratory fitness was related to better self-concept, less symptoms of depression and to lower levels of both state and trait anxiety. Furthermore, higher BMI was related to lower self-concept. Subsequent analysis on aspects of self-concept showed that better cardiorespiratory fitness was related to better perceived social acceptance, athletic competence, close friendships and self-esteem. Furthermore, better muscular fitness and speed-agility were related to better perceived athletic competence. Finally, higher body composition (BMI) was related to lower perceived physical appearance and self-esteem.

Previous studies on the relationship of physical fitness with psychosocial health mainly focused on overall measures of psychosocial functioning and depression. The current study extended these studies by including a wide range of specific psychosocial health outcomes including measures of self-concept, anxiety, and ADHD (Åvitsland et al., 2020, Bou-Sospedra et al., 2020, Rieck et al., 2013). Our results suggest that cardiorespiratory fitness is specifically related to lower levels of internalizing problems, as cardiorespiratory fitness was related to better self-concept, less symptoms of depression and to lower levels of both state and trait anxiety, but not to symptoms of ADHD, including inattentive and hyperactive-impulsive behavior. Several mechanisms might explain the observed inverse relationship between cardiorespiratory and muscular fitness and internalizing problems. One possibility is that better cardiorespiratory and muscular fitness enables easier access to and greater engagement in physical activity. Physical activity has been found to lead to higher concentrations of endorphins in the brain and to reduce activity of the sympathetic nervous system, in turn resulting in less internalizing problems in adults (Anderson and Shivakumar, 2013, Dinas et al., 2011, Mücke et al., 2018). Furthermore, engaging in physical activity can provide access to others also engaging in physical activities and support formation of meaningful relationships. Thus, both the biological mechanisms triggered by physical activity as well as building meaningful relationships might explain the observed relationship between physical fitness and internalizing problems. Finally, it could also be that the adolescents with more symptoms of internalizing problems are less willing to exercise and are therefore less fit (Bélair et al., 2018). Our study was a cross-sectional study and could not examine the causality of the studied relationship. Longitudinal and intervention studies are needed to provide evidence of causation.

Our results did not show any significant relationship between any domain physical fitness and symptoms of inattentive and hyperactive-impulsive behavior. This finding is not in line with the single available study in adolescents that investigated the relationship between physical fitness and ADHD symptoms and found that fit children are at decreased risk of developing ADHD symptoms (Muntaner-Mas et al., 2020). The few other studies investigating this relationship were all performed in typically developing children (Golubović et al., 2014) or young adults (Jeoung, 2014). These studies showed inconsistent results, with one study showing no relationships between the physical fitness levels and ADHD symptoms in both children with and without a diagnosis of ADHD (Golubović et al., 2014), and the other study showed that both low muscular strength and cardiorespiratory fitness were linked to increased ADHD symptoms in young adults (Jeoung, 2014). Perhaps the strongest evidence comes from intervention studies, which show that physical activity interventions have a positive effect on the symptoms of ADHD (Gapin et al., 2011, Suarez-Manzano et al., 2018, Cerrillo-Urbina et al., 2015). However, these studies were performed in children or adolescents diagnosed with ADHD and in most cases did not use blinded assessments which challenges the value of these studies. It might be argued that the beneficial effects of higher levels of all domains of physical fitness will only be evident in individuals with relatively high levels of ADHD symptoms, since these individuals will show most room for improvement. Converging evidence implicates dysregulation of the catecholaminergic circuits in the pathophysiology of ADHD with molecular imaging studies suggesting impaired neurotransmission due to abnormalities in the dopamine transporter (Doyle et al., 2005). Since physical fitness refers to the ability to engage in physical activity for a protracted period of time and physical activity is known to trigger the release of catecholamines, this in turn might remediate dysregulation of the catecholaminergic circuits resulting in lower levels of ADHD symptoms (Sonuga-Barke, 2005). We speculate that the beneficial effects of physical activity only become evident in those individuals with high levels of ADHD symptoms because in these individuals physical activity will normalize catecholaminergic neurotransmission, whereas in individuals showing no or only a few symptoms of ADHD, there will be no or only minor dysregulation of the catecholaminergic circuits and hence physical activity will not translate into changes in ADHD symptoms, if present. This might explain why in a healthy population the observed inverse relationship between physical fitness and ADHD symptoms is not revealed, whereas this relationship would become evident in clinical groups (Sonuga-Barke, 2005). This possibility might be investigated in future studies.

Another interesting result was that even after adjusting for cardiorespiratory fitness, muscular fitness and speed-agility, higher body composition (BMI) was related to lower self-concept in adolescents. Possibly this relationship between body composition and self-concept emerges because adolescents with higher BMI have more weight and shape concerns and might be faced with weight based stigmatization and teasing, in turn resulting in lower self-concept (Russell-Mayhew et al., 2012). Such an interpretation of the results further supported by the observed positive relationship between body composition and the aspects of self-concept including perceived physical appearance and self-esteem.

Next we examined the relationship between physical fitness and aspects of self-concept. Results indicate that cardiorespiratory fitness, muscular fitness and speed-agility were all found related to perceived athletic competence. This finding is in line with previous findings indicating that adolescents who perform well on physical fitness tests are also self-confident about their athletic competence (Jaakkola et al., 2019). Furthermore, cardiorespiratory fitness was significantly related to perceived social acceptance and close friendships. In their systematic review, Eime et al. suggested that adolescents who perform sports, more often develop friendships through their participation in sports, which in turn might have positive effects on perceived social acceptance and close friendships (Eime et al., 2013).

Another finding obtained in the analyses on aspects of self-concept was that none of the physical fitness outcomes was significantly related to the self-perception of school skills or behavioral attitude. This is somewhat surprising because earlier studies have suggested that higher cardiorespiratory fitness and overall physical fitness is related to better academic performance in adolescents (Chu et al., 2019). The discrepant findings might be related to the fact that previous studies have mostly focused on measures of academic performance, while the current study targeted self-perception of school skills or the behavioral attitude towards school work. Academic performance is mostly measured by language or mathematic tests, while the perceived competence of school skills is a perception of a student’s academic capabilities. The perceived capability is not only dependent of one’s objectively measured skills and knowledge, but also on one’s past subjective interpretation that might be related to past experiences or feedback of one’s teacher.

Finally, no significant relationships were found between muscular fitness and psychosocial problems. This result contrasts with findings in older adults where higher levels of muscular fitness were significantly related to reduced levels of psychosocial problems (O'Connor et al., 2010). The observed inverse relationship between muscular fitness and psychosocial problems in older adults is not surprising, considering how strength training improves daily functioning and facilitates social interactions, which in turn would benefit psychosocial health (Granacher et al., 2013). In adolescents, however, such a relationship between muscular fitness and psychosocial problems seems less plausible. It could be that low muscular fitness in adolescents reduces social interaction available through sports or activities, however, most adolescents seem not to be limited in their social interactions as a result of limited muscular fitness, as was the case in the population studied here.

### 4.1 Strengths and limitations

Strengths of the present study include the wide range of aspects of physical fitness and psychosocial health covered. Furthermore, this study used a representative group of adolescents recruited in both non-urban and urban regions and representing the full range of educational levels. A limitation of this study was the cross-sectional design that did not allow testing causal relationships.

## 5 Conclusion

Better cardiorespiratory fitness was associated with better self-concept and less symptoms of depression and anxiety. Furthermore, higher body composition was associated with lower self-concept in adolescents. The results suggest that better cardiorespiratory fitness and lower body composition might be a protective factor against psychosocial problems in adolescents and have a positive influence on self-concept. The current study emphasizes the importance of incorporating diverse aspects of physical fitness and psychosocial health in future studies into the relationship of physical fitness and psychosocial health.

### CRediT authorship contribution statement

**Barbara Franca Haverkamp:** Conceptualization, Methodology, Formal analysis, Investigation, Data curation, Writing – original draft. **Esther Hartman:** Conceptualization, Methodology, Writing – review & editing, Supervision. **Jaap Oosterlaan:** Conceptualization, Methodology, Writing – review & editing, Supervision.

## Declaration of Competing Interest

The authors declare that they have no known competing financial interests or personal relationships that could have appeared to influence the work reported in this paper.
