# Supplementary material for: Physical fitness and psychosocial health in a sample of Dutch adolescents
Source: Prev Med Rep. 2021 Dec 27;25:101689. doi: 10.1016/j.pmedr.2021.101689 (PMC8855700; doi:10.1016/j.pmedr.2021.101689)
Supplement: Supplementary data 1 [file mmc1.docx]

**Appendix**: Table A1. Results of multivariate multilevel regression analysis for the individual aspects of physical fitness and psychosocial health (n=361).

|  | Cardiorespiratory fitness | | | Muscular fitness | | | Speed- agility | | | | Body composition | | | |
| --- | --- | --- | --- | --- | --- | --- | --- | --- | --- | --- | --- | --- | --- | --- |
|  | B | SE | p | B | SE | p | B | SE | p | B | | SE | p |  |
| *Self-concept* |  |  |  |  |  |  |  |  |  |  | |  |  |  |
| Random intercept | -0.183 | 0.063 | 0.004 | 0.010 | 0.052 | 0.844 | 0.039 | 0.049 | 0.426 | 0.039 | | 0.048 | 0.422 |  |
| Sex^a^ | 0.323 | 0.092 | 0.000 | -0.033 | 0.066 | 0.620 | -0.075 | 0.064 | 0.238 | -0.076 | | 0.063 | 0.229 |  |
| VO2max | **0.290** | **0.046** | **0.000** |  |  |  |  |  |  |  | |  |  |  |
| Muscular fitness |  |  |  | **0.153** | **0.039** | **0.000** |  |  |  |  | |  |  |  |
| Speed-agility |  |  |  |  |  |  | **0.135** | **0.040** | **0.001** |  | |  |  |  |
| Body composition |  |  |  |  |  |  |  |  |  | **-0.131** | | **0.032** | **0.000** |  |
| Variance classes^b^ | 0.014 | 0.009 | 0.120 | 0.011 | 0.008 | 0.169 | 0.004 | 0.006 | 0.505 | 0.005 | | 0.006 | 0.405 |  |
| Variance adolescents^b^ | 0.326 | 0.025 | <0.001 | 0.350 | 0.027 | <0.001 | 0.361 | 0.027 | <0.001 | 0.356 | | 0.027 | <0.001 |  |
| *Depression* |  |  |  |  |  |  |  |  |  |  | |  |  |  |
| Random intercept | 0.235 | 0.104 | 0.024 | 0.004 | 0.086 | 0.964 | -0.017 | 0.084 | 0.838 | -0.017 | | 0.084 | 0.840 |  |
| Sex^a^ | -0.418 | 0.153 | 0.006 | 0.012 | 0.108 | 0.914 | 0.045 | 0.104 | 0.666 | 0.045 | | 0.104 | 0.663 |  |
| VO2max | **-0.334** | **0.076** | **0.000** |  |  |  |  |  |  |  | |  |  |  |
| Muscular fitness |  |  |  | **-0.144** | **0.064** | **0.025** |  |  |  |  | |  |  |  |
| Speed-agility |  |  |  |  |  |  | **-0.171** | **0.065** | **0.009** |  | |  |  |  |
| Body composition |  |  |  |  |  |  |  |  |  | **0.159** | | **0.052** | **0.002** |  |
| Variance classes^b^ | 0.033 | 0.019 | 0.082 | 0.031 | 0.018 | 0.085 | 0.026 | 0.017 | 0.126 | 0.029 | | 0.018 | 0.107 |  |
| Variance adolescents^b^ | 0.917 | 0.069 | <0.001 | 0.953 | 0.072 | <0.001 | 0.950 | 0.071 | <0.001 | 0.940 | | 0.071 | <0.001 |  |
| *State anxiety* |  |  |  |  |  |  |  |  |  |  | |  |  |  |
| Random intercept | 0.083 | 0.098 | 0.397 | -0.088 | 0.079 | 0.266 | -0.093 | 0.078 | 0.230 | -0.085 | | 0.077 | 0.272 |  |
| Sex^a^ | -0.155 | 0.155 | 0.319 | 0.164 | 0.110 | 0.136 | 0.173 | 0.106 | 0.103 | 0.157 | | 0.105 | 0.135 |  |
| VO2max | **-0.226** | **0.078** | **0.004** |  |  |  |  |  |  |  | |  |  |  |
| Muscular fitness |  |  |  | -0.035 | 0.065 | 0.595 |  |  |  |  | |  |  |  |
| Speed-agility |  |  |  |  |  |  | -0.038 | 0.066 | 0.563 |  | |  |  |  |
| Body composition |  |  |  |  |  |  |  |  |  | **0.105** | | **0.053** | **0.045** |  |
| Variance classes^b^ | 0.000 | 0.000 | 1.000 | 0.000 | 0.000 | 1.000 | 0.000 | 0.000 | 1.000 | 0.000 | | 0.000 | 1.000 |  |
| Variance adolescents^b^ | 0.965 | 0.072 | <0.001 | 0.988 | 0.074 | <0.001 | 0.987 | 0.074 | <0.001 | 0.977 | | 0.073 | <0.001 |  |
| *Trait anxiety* |  |  |  |  |  |  |  |  |  |  | |  |  |  |
| Random intercept | -0.044 | 0.095 | 0.641 | -0.221 | 0.076 | 0.004 | -0.250 | 0.075 | 0.001 | -0.242 | | 0.075 | 0.001 |  |
| Sex^a^ | 0.083 | 0.151 | 0.584 | 0.411 | 0.106 | 0.000 | 0.466 | 0.103 | 0.000 | 0.451 | | 0.102 | 0.000 |  |
| VO2max | **-0.267** | **0.075** | **0.000** |  |  |  |  |  |  |  | |  |  |  |
| Muscular fitness |  |  |  | **-0.137** | **0.063** | **0.030** |  |  |  |  | |  |  |  |
| Speed-agility |  |  |  |  |  |  | -0.058 | 0.064 | 0.368 |  | |  |  |  |
| Body composition |  |  |  |  |  |  |  |  |  | **0.121** | | **0.051** | **0.017** |  |
| Variance classes^b^ | 0.000 | 0.000 | 1.000 | 0.000 | 0.000 | 1.000 | 0.000 | 0.000 | 1.000 | 0.000 | | 0.000 | 1.000 |  |
| Variance adolescents^b^ | 0.906 | 0.068 | <0.001 | 0.926 | 0.069 | <0.001 | 0.936 | 0.070 | <0.001 | 0.924 | | 0.069 | <0.001 |  |
| *Inattentive behavior* | | | | | | | | | | | | | |  |
| Random intercept | -0.085 | 0.104 | 0.414 | -0.040 | 0.085 | 0.640 | -0.041 | 0.084 | 0.624 | -0.046 | | 0.084 | 0.585 |  |
| Sex^a^ | 0.151 | 0.155 | 0.330 | 0.069 | 0.109 | 0.524 | 0.066 | 0.105 | 0.528 | 0.080 | | 0.105 | 0.443 |  |
| VO2max | 0.054 | 0.078 | 0.490 |  |  |  |  |  |  |  | |  |  |  |
| Muscular fitness |  |  |  | -0.006 | 0.064 | 0.923 |  |  |  |  | |  |  |  |
| Speed-agility |  |  |  |  |  |  | -0.018 | 0.065 | 0.785 |  | |  |  |  |
| Body composition |  |  |  |  |  |  |  |  |  | -0.029 | | 0.052 | 0.580 |  |
| Variance classes^b^ | 0.025 | 0.024 | 0.298 | 0.024 | 0.024 | 0.317 | 0.024 | 0.024 | 0.317 | 0.026 | | 0.024 | 0.279 |  |
| Variance adolescents^b^ | 0.974 | 0.074 | <0.001 | 0.976 | 0.075 | <0.001 | 0.978 | 0.075 | <0.001 | 0.975 | | 0.074 | <0.001 |  |
| *Hyperactive and impulsive behavior* | | | | | | | | | | | | | |  |
| Random intercept | 0.009 | 0.112 | 0.939 | -0.026 | 0.093 | 0.782 | -0.054 | 0.093 | 0.558 | -0.064 | | 0.093 | 0.493 |  |
| Sex^a^ | -0.002 | 0.157 | 0.988 | 0.063 | 0.107 | 0.559 | 0.115 | 0.104 | 0.270 | 0.132 | | 0.104 | 0.205 |  |
| VO2max | -0.099 | 0.079 | 0.208 |  |  |  |  |  |  |  | |  |  |  |
| Muscular fitness |  |  |  | **-0.173** | **0.064** | **0.007** |  |  |  |  | |  |  |  |
| Speed-agility |  |  |  |  |  |  | **-0.147** | **0.066** | **0.027** |  | |  |  |  |
| Body composition |  |  |  |  |  |  |  |  |  | 0.063 | | 0.052 | 0.227 |  |
| Variance classes^b^ | 0.061 | 0.036 | 0.090 | 0.058 | 0.035 | 0.097 | 0.062 | 0.036 | 0.085 | 0.062 | | 0.036 | 0.085 |  |
| Variance adolescents^b^ | 0.938 | 0.072 | <0.001 | 0.927 | 0.071 | <0.001 | 0.929 | 0.071 | <0.001 | 0.937 | | 0.072 | <0.001 |  |

*Note*. ^a^Boys was the reference category; ^b^These values represent respectively the between and within class variance
